# Desquamative Nephritis

**Published:** 1853-09

**Authors:** 


From the Ohio Med. and Surg. Journal.

*Desquamative Nephritis.*

The following is an extract of a letter from our highly esteemed friend, Dr. J. Bassett Chapin, now a resident of the New York Hospital. The case described and the remarks upon it, are very interesting, and will be read with profit by every one. We hope hereafter to receive similar favors.—*Ed. O. Med. & Surg. Jour.*

* * * In no department of investigation has greater progress been effected than in the study of Renal diseases. To Dr. George Johnson, of London, is due the credit of clearing away the veil that has long obscured these diseases, who has demonstrated the necessity of the microscope for their proper diagnosis. He divides all kidney affections, attended with albumen in the urine, into two varieties; the first he denominates Desquamative Nephritis, embracing that variety of disease characterized by the presence in the urine of eqitherial cells, and fibrinous casts of the uriniferous tubules, and often blood. The second variety, by the presence of epithelial cells, waxy colored casts, and fat globules. These globules exist in the cells, and load the casts, or by their abundance, rupture the cells, and exist free in the urine. Blood is often an accompaniment to the above throughout the disease; this is the true granular disease of Bright—a fatty degeneration of the kidney.

Since the announcement of the above results, great interest has been excited to see how far repeated observation would tend to confirm them. The cases of albuminous urine have been attentively observed the past year by Dr. Van Arsdale, the microscopist of this hospital; and I am sure I can write you nothing of more interest than to communicate to you the history and full notes of a case most recently under treatment. This, you will observe, confirms fully the characteristics of the first variety; and I select this from several on account of many features of interest involving the treatment pursued.

Case.—A German, aged 36, a farmer, was admitted April 12th, during the attendance of Dr. Swett. Stated he enjoyed good health till five weeks ago, when he arrived in an emigrant vessel from Liverpool. About this time attacked with dizziness, vomiting and diarrhœa, together with considerable febrile excitement; but does not recollect to have had any sore throat, rash, or eruption over body; was convalescent in three weeks, marked by return of appetite and strength. Three weeks ago noticed an œdematous swelling appearing first in the legs, then in face, and extending to abdomen and body generally. About this time noticed his urine very high colored, and was obliged to void it often during the night. Has complained of no pain about region of kidneys.

At present, pulse 100, good strength, skin cool and *dry*, tongue natural, bowels regular, appetite fair. Legs and abdomen considerably distended, face puffy. There appears to be a general exfoliation of the cuticle over body, and spots of purpura. No tenderness over kidneys on pressure. Complains that he is very much affected by changes of temperature.

On examination of liver and heart, no enlargement detected, apex strikes natural; faint blowing sound, with sound of heart, over aortic valves.

Quantity of urine in 24 hours, two pints, very high colored, frothy, of acid reaction. Sp. gr. 1006, depositing, by heat, and nitric acid, an almost solid deposit of albumen.

Microscopic examination shows urine to contain blood in moderate quantity, epithelial cells, and casts of the uriniferous tubes. No fat globules observed.

C. C. ad reg. lumb.

℞. Hot air bath, ter in die.

℞. Ipecac pulv. gr. ¼.

Mind. Spiritus ℥ss. M. 9. 4. h.

April 15. Urine 3 pints in quantity, Sp. gr. 1004. Is clearer, contains much less albumen. Skin becomes moist during application of hot air, but generally dry. Has had two days looseness of bowels, stools contain mucus and slime.

April 17. Urine last 24 hours, 6½ pints, Sp. gr. 1003, dark colored, shows but a faint cloud of albumen by chemical agents. Microscopic examination, shows presence of cells in abundance, no blood, no casts. Perspired freely for two hours last evening. Dysenteric stools continued.

Discont. above.

℞. Pulv. Dov. grs. v. p. r. n.

April 19. Quantity of urine last 24 hours, 6¾. Sp. gr. 1004. Dropsy has very much diminished. Has had 6 stools last 24 hours, containing blood. Skin acts freely.

℞. Opii, P. grs. ij.

Plumb. Act. grs. ij H. Pill. 9. 4. h.

April 21. Quantity same. Sp. gr. 1004. Microscope shows a little blood, a few epithelial cells, no casts. Skin perspiring freely. Dysentery checked.

Discont. above.

C. C. ad reg. lumb.

℞. Hot air bath.

℞. Spts. Mind. et Ipecac. Mist. 9. 4. h.

April 25. Quantity of urine 5½ pints. Sp. gr. 1005, of dark color. Microscope shows it to contain cells and fibrinous casts in much less quantity. Skin acts freely ; dropsy subsiding ; not any about face or abdomen ; most marked about ankles.

May 9. The quantity of urine has been gradually increasing, reaching 12 pints. Sp. gr. 1004, and much clearer.

Chemical examination shows the slightest possible trace of albumen. Microscope shows nothing but mass of disintegrated cells.

General condition improving, and walks about the ward for first time.

Resolved to discontinue all treatment.

May 25. From time of last note, urine has been gradually diminishing, amounting last 24 hours to 5 pints. Sp. gr. 1006. No albumen ; nothing microscopic ; very slight œdema about ankles.

Walks about ; appetite good.

June 5. Quantity of urine, 3½ pints. Sp. gr. 1008. Is considered to be in a condition to be discharged at the expiration of his time.

You will observe the disease run its course to a favorable termination ; there were many reasons to suppose the patient had contracted scarlatina from his exposure on ship-board, and from the exfoliating process going on in the skin, though no other points were elicited.

The microscope showed the same desquamative process going on in the glandular uriniferous tubes, the abundance of their cells, their subsidence, and final disappearance.

To explain the phenomenon of the above, it is necessary to recall the anatomy of the kidney as demonstrated by Todd and Bowman, and to know that the uriniferous tubes are lined by glandular epithelial cells, resting upon a basement membrane, whose office is to eliminate the solid constituents of the urine.

The pathology of these appearances depends upon the fact, that the kidneys take upon themselves to eliminate from the blood foreign matter that may be existing in it, as when the exfoliation of the cuticle, after scarlatina is arrested, the kidneys eliminate what is supposed to be the "*materies morbi*;" to perform this function, the death of the cell and its presence in the urine, is the result, which is always to be looked upon with suspicion ; for no cell ought normally to exist in the urine.

This desquamative process may go on then according to the condition of the blood, new cells produced ; but if it is to continue, become chronic, a time will come when they will lose their primitive vitality, none be reproduced, and atrophy from disease, and permanent disease result.

The presence of blood corpuscles, and, of albumen, is owing to congestion of the malphighian bodies, and mechanical exudation ; the fibrin coagulating furnishing the casts of the tubes, which are washed away in the flow of urine existing generally through the course of the disease.

These diseases generally run their course to a favorable termination, leaving no permanent disease of the kidney; properly to diagnosticate this variety, and especially as aiding us in the prognosis, the microscope is to become an indispensable agent.

In the treatment of this disease, the microscope indicates rather a negative course. The cells, as they fall off, very often block up the tubes, and it would be obvious that diuretics, or substances likely to irritate the kidneys, would increase the difficulty. The indication is to relieve the congestion of the kidneys, manifested by the presence of blood in the urine; this, perhaps, ought to be received with some caution; for, in some cases, the hemorrhage seemed to be rather a passive one than active.

To relieve the dropsical accumulations, different plans are suggested; but the treatment pursued here and oftener successful, perhaps, is the diaphoretic. In reviewing the above case, you will observe the result. The patient received internally the Spiritus Mind. and Ipecac., and externally was administered the hot air bath for 30 minutes at a time, repeated during the day. In three days the skin, hitherto dry and harsh, became relaxed, and in five days diaphoresis was established, much to the relief of the kidneys.

When the skin commenced to act, the urinary secretion began to increase, and the bowels were spontaneously loosed.

I have endeavored, as briefly as possible, to detail the case above, and hope it will prove of sufficient interest to pay a perusal.

